# Roadblocks in the Path of iPSC to the Clinic

**DOI:** 10.1007/s40472-018-0177-x

**Published:** 2018-02-08

**Authors:** Elena Garreta, Sonia Sanchez, Jeronimo Lajara, Nuria Montserrat, Juan Carlos Izpisua Belmonte

**Affiliations:** 1 Pluripotent stem cells and activation of endogenous tissue programs for organ regeneration (PR Lab), Institute for Bioengineering of Catalonia (IBEC), The Barcelona Institute of Science and Technology, Baldiri Reixac 10-12, 08028 Barcelona, Spain; 2 Universidad Católica San Antonio de Murcia (UCAM), Campus de los Jerónimos, 135 Guadalupe, 30107 Murcia, Spain; 3 Networking Biomedical Research Center in Bioengineering, Biomaterials and Nanomedicine (CIBER-BBN), Madrid, Spain; 4 Gene Expression Laboratory, Salk Institute for Biological Studies, 10010 North Torrey Pines Road, La Jolla, CA 92037 USA

**Keywords:** Human embryonic stem cells, Induced pluripotent stem cells, Induced pluripotent stem cells, Immunogenicity

## Abstract

**Purpose of Review:**

The goal of this paper is to highlight the major challenges in the translation of human pluripotent stem cells into a clinical setting.

**Recent Findings:**

Innate features from human induced pluripotent stem cells (hiPSCs) positioned these patient-specific cells as an unprecedented cell source for regenerative medicine applications. Immunogenicity of differentiated iPSCs requires more research towards the definition of common criteria for the evaluation of innate and host immune responses as well as in the generation of standardized protocols for iPSC generation and differentiation. The coming years will resolve ongoing clinical trials using both human embryonic stem cells (hESCs) and hiPSCs providing exciting information for the optimization of potential clinical applications of stem cell therapies.

**Summary:**

Rapid advances in the field of iPSCs generated high expectations in the field of regenerative medicine. Understanding therapeutic applications of iPSCs certainly needs further investigation on autologous/allogenic iPSC transplantation.

## Introduction

**Fig. 1:**
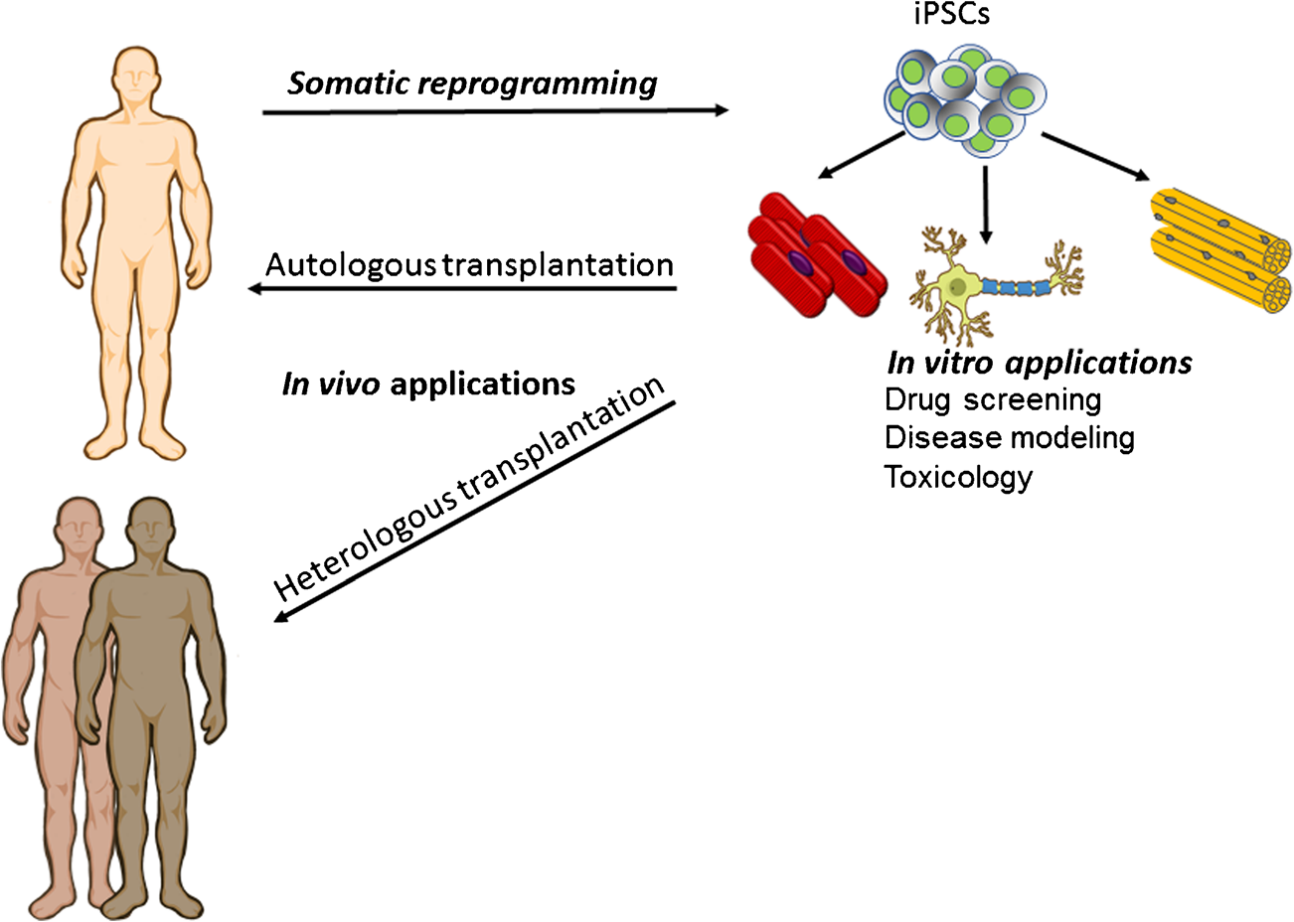
The generation of hiPSCs from different somatic cell sources has opened a *plethora* of in vitro applications in patient-hiPSCs derivatives. More importantly, current efforts are guided towards the generation of HLA-matched iPSCs by genome editing. Other alternatives for further application of iPSCs into the clinics may involve the generation of allogeneic iPSCs cell banks worldwide

In the next few months, the seminal discovery that human embryonic stem cells (hESCs) could be derived from pre-implantation embryos and indefinitely expanded in vitro will celebrate its 20th anniversary [1]. This landmark finding generated high hopes, positioning hESCs as a major cell source for Regenerative Medicine purposes. hESCs were rapidly considered to be a ready-to-use cellular product with the potential to give rise to any cell type belonging to the three germ layers of the embryo (i.e., ectoderm, mesoderm, or endoderm derivatives). With the goal of transferring hESCs into the clinical arena, the scientific community developed initial protocols for hESC differentiation towards the major organ systems compromised in human diseases (e.g., neurons, cardiomyocytes, and hematopoietic stem cells, among others). However, despite all these initial expectations, two major roadblocks preclude hESC translation to the clinical setting. On one side there are the ethical considerations related to the use of human embryos for their derivation, and on the other side are issues related with immune rejection after transplantation (Fig. 1).

All these concerns were solved in 2006 and 2007, when Takahashi and Yamanaka first described that the pluripotent state found in in vitro captured hESCs could be artificially induced by the overexpression of just four transcription factors (OCT4, SOX2, cMYC, and KLF4-OSKM) [2]. The resulting cells, so-called induced pluripotent stem cells (iPSCs), exhibited all the molecular and functional features of hESCs while circumventing the ethical problems associated with the use of human embryos and immune rejection after transplantation. Importantly, it is worthy to mention that the history of somatic reprogramming was initially written almost 60 years ago [3]. By that time Gurdon and colleagues, using the technique of somatic cell nuclear transfer (SCNT) showed that the nuclei of intestinal epithelial cells from *Xenopus leavis* if transplanted into enucleated eggs could develop into normal and healthy tadpoles, demonstrating successful nuclear reprogramming [3]. Later, one of the most important advances in the field was the birth of the first cloned mammal, Dolly the sheep, in 1998 [4]. In the last two decades, progress has been made producing “clones” for reproductive purposes in several species—cattle, goats, mice, and pigs [5–11] culminating this period with the creation of the first cloned human embryo in 2013 by the group of Mitalipov [12]. Nevertheless, mouse ESCs generated by SCNT (ESCs-SCNT) retain the mitochondria from the recipient oocyte, which induces alloimmunity after transplantation in mice genetically matched to the reprogrammed nucleus [13].

Since iPSC technology was first applied in humans in 2007, it has generated great expectations in the field, as this new type of patient-specific cell shares with human ESCs (hESCs) the capacity to indefinitely self-renew while preserving pluripotency-related features. In the last 10 years, patient-specific iPSCs have represented the Holy Grail for Regenerative Medicine applications, as well as for the fields of disease modeling and drug discovery [14]. However, despite the overall general excitement and push to move things forward quickly, the stem cell field has also imposed high standards onto hiPSCs. Soon after their initial discovery, their value as a faithful disease model and autologous source of safe cells has been intensely debated. Indeed, in most of the cases the same scientists that made extensive progress in the iPSC field have been the ones questioning their translation into the clinic over the last 10 years. In this regard, genetic mutations and chromosomal aberrations detected in iPSCs generated from different cell sources and utilizing different methodologies have raised concerns about their tumorigenic potential [15]. Likewise, the detection of epigenetic aberrations have put into question the differentiation potential of iPSCs and immune tolerance after autologous transplantation [16]. Again, collaborative efforts have demonstrated that these divergences were mostly due to technical restrictions that have been elegantly solved over the years by important advances in the stem cell field. In this regard, the stem cell research community has produced safe methods for the generation and expansion of human pluripotent stem cells (hPSCs-both iPSCs and hESCs). Overall, all these improvements reveal the crescent awareness in translating hPSC applications into a clinical setting.

In this review article, we evaluate the technical and practical obstacles to the clinical translation of iPSCs. We will also comment on preclinical features that should be addressed before iPSC transplantation, such as inherent tumorigenic potential and problems arising from their differentiation into heterogeneous mature adult types. In the last part, we will briefly cite further considerations for their efficient clinical implementation, such as common criteria to be used for autologous/allogenic iPSC transplantation.

## Immune Response Evaluation in Autologous iPSCs

Since their discovery, patient-specific iPSCs have been considered theoretically safe in the setting of autologous transplantation. These assumptions positioned iPSCs as the ideal source for transplantation purposes, since patients would avoid live-long immunosuppressive treatment for the prevention of allograft rejection [17]. However, this affirmation became highly questioned when Zhao and colleagues described that when transplanted in syngeneic hosts, iPSCs, but not ESCs, could led to T cell infiltration, tissue necrosis, and immunogenic teratomas [18]. Furthermore, the immune rejection response observed by the authors was totally T cell dependent, since it was blocked in Rag knock-out recipients. Likewise, since only iPSC-derived teratomas expressed a subset of residual antigens compared to ESCs, the authors concluded that these findings were due to the incomplete reprogramming of iPSCs [18]. These surprising findings opened intense debate in the field, and several important journals commented on the possible failure of translating iPSCs into the clinic, lowering initial expectations. Again, and due to the importance of these results, other works tried to shed light on to the Zhao observations. For example, Araki and colleagues [19] bypassed the issue of incomplete differentiation by transplanting in vivo-differentiated tissues from iPSC-chimeric mice into genetically matched recipients. By this approach, the authors showed that in their experimental setting iPSC transplantation elicited limited immunogenicity, concluding that iPSC derivatives do not cause an immune response [19]. Nevertheless, in the same work, ectopically transplanted iPSC-derived cardiomyocytes evoked a T cell immune response in syngenic mice, but not when iPSCs were differentiated through in vivo chimera formation [19].

From the very first moment after their discovery, patient-iPSC derivatives have been considered the main cell source for autologous cell replacement therapies in front of other pluripotent sources (hESCs derived by SCNT, parthenogenetic hESCs among others). In this regard, common efforts have been employed towards evaluating the immunogenicity of in vitro iPSC-derived cells. Towards this end, Guha and collaborators [20] recently differentiated syngeneic iPSCs into embryoid bodies or representative cell types from each of the three embryonic germ layers (endothelial cells, hepatocytes, and neuronal cells) and transplanted them into the subcapsular renal space of syngenic recipients [20]. In their experimental setting, the authors did not observe an immune response and concluded that autologous iPSC derivatives do not elicit an immunogenic response [20]. Although these findings arrived during a very controversial time in the iPSC field, the scientific community still continued posing questions about iPSCs immunogenicity. Moreover, these observations contradicted previous work from Araki and colleagues, and most importantly, did not reflect a true therapeutic scenario, in where functional iPSC derivatives would be injected at the site of interest [17]. In this regard, other studies have further investigated, side-by-side, the immune response of parental iPSCs versus iPSC-derived counterparts and endogenous isolated endothelial cells when transplanted in isogenic hosts [21]. Interestingly, the pioneering work by de Almeida and colleagues revealed that none of the differentiated cell types used in their study provoked an immune response [21]. The most important finding of this study was that T cells attracted to either iPSC-derived endothelial cells or in vivo derived controls were indistinguishable from each other, but distinct from T cell clones which infiltrate iPSC grafts, as demonstrated by single-cell qPCR comparative analysis. Altogether, this work suggested that the immune response to undifferentiated iPSCs is different from their mature derivatives, and highlights the need to perform similar comparative analyses in starting cell populations in order to ascertain the extent to which the degree of differentiation correlates with immune tolerance after transplantation [17]. In this regard, Zhao and colleagues have recently shown that, taking advantage of humanized mouse models, it is possible to test for the immunogenicity of autologous cells derived from hiPSCs [22]. Interestingly, this study showed that whereas autologous hiPSC-derived-smooth muscle cells were highly immunogenic, autologous hiPSC-derived retinal pigment epithelial (RPE) cells were immune tolerated, regardless of the injection site [22].

Other works along this line have also proved the immunogenic potential of iPSCs in non-human primates where autologous iPSC-derived dopamine neurons implanted into the brain showed lasting engraftment and tolerance in the absence of immunosuppression for 2 years [23, 24], while in another study, they were reported to provoke only a minimal immune response compared with allogeneic cells [25].

Overall, these results highlight the necessity to define the initial variations between undifferentiated iPSC clones that upon differentiation may trigger different degrees of inflammation when implanted into syngeneic recipient animals. Such alterations may arise from disparities already found in the initial cells to be reprogrammed, leading to incomplete reprogramming. Similarly, epigenetic variations between the iPSC clones derived from the same donor may also predispose them to differences in differentiation outcomes. In this regard, further comparative analyses from undifferentiated iPSC clones versus their derived counterparts at different stages during the differentiation process, would help to detect the amount of gene expression differences that can be tolerated by the immune system after transplantation. Likewise, the establishment of standardized protocols for iPSC differentiation may take advantage of strategies aimed at identifying and suppressing partially differentiated cells at the end of the differentiation process. In this regard, the use of genome editing technologies represent a powerful tool for the generation of hPSC reporter cell lines where single or dual fluorescent reporters would help to select for desired or unwanted cell types using fluorescence-activated cell sorting.

## Considerations for iPSC Biobanking

Recent findings on the autoreactive immune response to iPSCs or their mature derivatives would suggest a need for a degree of immunosuppression [26]. In this regard, it has been already accepted that the generation of standardized stem cell banks of iPSCs with genetically-defined MHC/human leucocyte antigen (HLA) molecules may prove valuable. Importantly, the generation of allogeneic iPSC banks could also significantly reduce the cost for iPSC-based cell therapy [14]. Recently, Nakatsuji and colleagues have presented a comprehensive study for the estimation of the scale of iPSC banking required to provide adequately matched iPSC lines in Japan. In their study, the authors concluded, after screening a database of 24,000 people, that it would be possible to generate 50 homozygous iPSC lines with a haplotype match for 90.7% of the Japanese population at HLA-A, HLA-B, and HLA-DR loci with two-digit specification [27]. These findings were confirmed in successive studies, which showed that 150 homozygous cell lines could provide a haplotype match for 93% of the population of the UK [28, 29••]. Other groups have shown that generating a master cell bank for more diverse populations would be more challenging. In this regard, using a probabilistic model it has been recently estimated that 22,000 individuals of European descent would have to be screened to generate 17 iPSC lines to offer a haplotype match to approximately 50% of that patient population [30]. A screen of 10,000 random individuals in the same North American population would offer a haplotype match to 45% of Hispanics, 35% of Asian Americans, and 22% of African Americans. Similarly, an iPSC bank of the 100 most common HLA types population wide would offer a haplotype match to 78% of individuals of European origin, 63% of Asians, 52% of Hispanics, and 45% of African Americans, suggesting that customized banking for each ethnic group does not necessarily solve this problem. Hence, an allogeneic cell bank in genetically homogenous countries, like Japan or Iceland for example, could be a more viable option, whereas a similar bank in the USA may be cost prohibitive due to the genetic heterogeneity in the country.

## Future Directions of Research

Currently, only a few clinical trials are in progress for hPSC cell derivatives. Specifically, as revised recently [14], out of the 13 ongoing clinical trials evaluating stem cell therapy products, eight are for hESC- and one is for hiPSC-derived RPE to treat macular degeneration. Indeed, in 2014, the first clinical study using human iPSC-derived RPE was initiated; however, it was subsequently put on hold in March 2015, owing to mutations observed in a second transplanted patient. Interestingly, the first data published from this clinical trial a few months ago also indicates no evidence of immune rejection 1 year after the transplantation of iPSC-derived RPE cell sheets under the retina in the first patient [31••].

Recently, the use of allogeneic iPSCs for therapeutic applications has been proposed, especially for those conditions where the cellular product may function in an immunoprotective environment [14]. In this regard, the generation of allogenic iPSC banks would reduce cost and regulatory barriers, two major roadblocks when translating iPSC technology to the clinic. All these improvements will soon benefit from the use of genome editing tools for the generation of “universal” iPSCs with better tolerance capacities upon transplantation [32••]. The resolution of ongoing clinical trials using human pluripotent stem cells over the next 2 to 3 years together with all these advances could finally result in exciting new findings in the decades to come, and thus provide fundamental knowledge on either autologous or allogenic iPSC translation into a clinical setting.

## Abbreviations

hESCs: Human embryonic stem cells
iPSCs: Induced pluripotent stem cells
SCNT: Somatic cell nuclear transfer
HLA: Human leucocyte antigen
RPE: Retinal pigment epithelial
